# Association between depression and anxiety on symptom and function after surgery for lumbar spinal stenosis

**DOI:** 10.1038/s41598-022-06797-1

**Published:** 2022-02-18

**Authors:** U. Held, J. M. Burgstaller, M. Deforth, J. Steurer, G. Pichierri, M. M. Wertli

**Affiliations:** 1 Department of Biostatistics at Epidemiology, Biostatistics and Prevention Institute, University of Zurich, Hirschengraben 84, 8001 Zurich, Switzerland; 2 Department of Internal Medicine, Horten Centre for Patient Oriented Research and Knowledge Transfer, University of Zurich, Pestalozzistrasse 24, 8032 Zurich, Switzerland; 3 Institute of Primary Care, University and University Hospital Zurich, Pestalozzistrasse 24, 8032 Zurich, Switzerland; 4 Department of General Internal Medicine, Kantonsspital Baden, Im Ergel 1, 5404 Baden, Switzerland; 5 Division of General Internal Medicine, Bern University Hospital, University of Bern, Freiburgstrasse 16p, 3010 Bern, Switzerland

**Keywords:** Medical research, Outcomes research

## Abstract

Evidence on the role of depression and anxiety in patients undergoing surgical treatment for symptomatic degenerative lumbar spinal stenosis (DLSS) is conflicting. We aimed to assess the association between depression and anxiety with symptoms and function in patients undergoing surgery for DLSS. Included were patients with symptomatic DLSS participating in a prospective multicentre cohort study who underwent surgery and completed the 24-month follow-up. We used the hospital anxiety and depression scale (HADS) to assess depression/anxiety. We used mixed-effects models to quantify the impact on the primary outcome change in the spinal stenosis measure (SSM) symptoms/function subscale from baseline to 12- and 24-months. Logistic regression analysis was used to quantify the odds of the SSM to reach a minimal clinically important difference (MCID) at 24 months follow-up. The robustness of the results in the presence of unmeasured confounding was quantified using a benchmarking method based on a multiple linear model. Out of 401 patients 72 (17.95%) were depressed and 80 anxious (19.05%). Depression was associated with more symptoms (β = 0.36, 95% confidence interval (CI) 0.20 to 0.51, p < 0.001) and worse function (β = 0.37, 95% CI 0.24 to 0.50, p < 0.001) at 12- and 24-months. Only the association between baseline depression and SSM symptoms/function was robust at 12 and 24 months. There was no evidence for baseline depression/anxiety decreasing odds for a MCID in SSM symptoms and function over time. In patients undergoing surgery for symptomatic DLSS, preoperative depression but not anxiety was associated with more severe symptoms and disability at 12 and 24 months.

## Introduction

Degenerative lumbar spinal stenosis (DLSS) is a common indication for spine surgery in in the elderly^1,2^. In symptomatic patients, the narrowing of spinal canal and compression of neural roots result in neurogenic claudication, a pain in the gluteal region and/or lower extremity during walking, which relieves through rest and lumbar flexion; with or without low back pain^3,4^. Pain and functional limitations results in a strong negative influence on health-related quality of life^5^. In symptomatic patients, treatment options include watchful waiting, physiotherapy, pain medications, injection of analgesics and steroids, and decompression surgery^6^.

Although surgery has been shown to be effective, not all patients will benefit from the treatment^7^. Approximately one-third of patients undergoing surgery will eventually not achieve a clinically relevant improvement^7^. Further, imaging studies in asymptomatic adults aged 60 years or older showed in up to 20% a stenosis without pain^8^. Various factors may influence treatment success in elderly patients. Age, sex, and comorbidities have been shown to result in longer hospital stay and more peri- and postoperative complications^9–12^. Psychological factors such as depression and anxiety may be also relevant. Depression has been shown to negatively influence treatment efficacy in patients with DLSS^13^. The evidence on anxiety and the interaction between anxiety and depression is less well studied and conflicting^14,15^.

The aim of this study is to evaluate whether depression and/or anxiety at baseline are associated with symptom severity and functional disability in patients with DLSS undergoing surgery. A novel methodological approach was used to assess the robustness of the results in the presence of unmeasured confounding^16^.

## Methods

### Data source and patient selection

Secondary analysis of patients included in a multicentre prospective cohort study conducted in eight hospitals in German-speaking Switzerland. The details of the study have been described previously^3,17–19^. In summary, patients were included into the Lumbar Stenosis Outcome Study (LSOS) if they (1) were ≥ 50 years old, (2) suffered from neurogenic claudication, (3) had a clinically and radiologically verified diagnosis of DLSS, (4) had a life-expectancy of > 1 year, (5) provided informed consent, and (6) follow-up assessment were feasible. Excluded were patients requiring urgent surgery, acute fractures or infection, excessive lumbar scoliosis (> 15 degrees), verified peripheral arterial disease^19^. For the current study, we included patients who underwent surgery up to 6 months of study inclusion and completed a 24-month follow-up. Excluded were patients who underwent non-surgical treatments.

The study was approved by the ethical committee (canton Zurich, Switzerland, KEK-ZH-NR: 2010-0395/0). The study was reported according to the STROBE guidelines^20^.

### Surgical procedure

The choice of the surgical procedures was at the discretion of the treating physician. Procedures included decompression surgery alone or with fusion techniques. Decompression surgery consisted of a standard open posterior lumbar decompression of affected level(s) with or without decompression of the lateral recessus and the foramina where appropriate. Fusion surgery consisted of additional implantation of pedicle screws with rods, and intersomatic fusion and cage(s) at the affected level(s). All procedures were performed or supervised by senior orthopaedic/neurosurgeons (> 10 years of experience after board certification).

### Depression and anxiety definition

Anxiety and depressive symptoms were assessed using the self-rated, validated, and reliable hospital anxiety and depression scale (HADS)^21^. The HADS consists of an anxiety and a depression subscale with a score-range of 0 (best) to 21 (worst) for each subscale^22,23^. Patients were categorized as depressed if the score was ≥ 8 points on the HADS depression subscale (HADS-D)^21^. Anxiety was defined as ≥ 8 points on the HADS anxiety subscale (HADS-A)^21^. Sensitivity and specificity of a cut-off of ≥ 8 points for both HADS subscales to identify patients with depression/anxiety has been shown to be between 0.70 and 0.90^21^.

### Outcomes

The primary outcomes were symptoms (pain) and physical function assessed using the spinal stenosis measure (SSM) symptoms and function subscale at 12 and 24 months. Secondary outcomes were meaningful clinically important difference (MCID) in the SSM symptoms and function at 24 months, complications, and reoperation rates. The SSM German version is a reliable and validated brief self-administered questionnaire (Supplementary Appendix 1)^24,25^. The symptom subscale ranges from 1 to 5 points (best–worst) and the function subscale from 1 to 4 (best–worst). The MCID in the SSM symptoms subscale was a 0.48 point improvement (decrease) and in the SSM function subscale score 0.52 points^24,25^. In patients with baseline values below the thresholds (SSM symptoms < 1.48, SSM function < 1.52), and remaining low SSM scores at all time points, an MCID of 1 was imputed at 24 months follow-up.

### Sample size justification

The number of patients available for this study was large enough for fitting multiple linear mixed-effects regression models with a time trend, including the parameters of interest (depression and anxiety) as well as eight other baseline characteristics to adjust for confounding.

### Confounder variables

Patients completed a set of self-reported questionnaires at baseline and additional information on comorbidities and smoking were collected during a structured interviews to complete the Cumulative illness rating scale (CIRS)^26^. Each patient was asked whether he/she was currently smoking. In case a patient was a non-smoker, we additionally asked for smoking in the past. Smoker was defined as a patient that reported currently to smoke. Low back and buttock pain were assessed in self-reported questions at the baseline evaluation.

### Statistical analysis

Descriptive statistics included mean and standard deviation (SD) (continuous/ordinal variables) and counts and percentages (categorical variables). Groups (depression/no depression and anxiety/no anxiety) were compared with exploratory p-values from χ^2^ and t tests. A predefined set of confounders^18^ was used including age, gender, body mass index (BMI) (≥ 25 kg/m^2^), civil risk (living alone, or single/divorced/widowed and living in a nursing/residential home), duration of symptoms (> 6 months), cumulative illness rating scale (CIRS), epidural injections within 90 days before baseline, and necessity of fusion surgery. Time was additionally included as a categorical variable, to account for overall trends at 12 and 24 months. We used multiple linear mixed effects regression models with continuous SSM symptom and function scores (separately) over time. Repeated observations in SSM symptoms and function scores were captured in patient-specific random intercept terms. The effects of depression and anxiety were adjusted for overall change from baseline to 12 and 24 months in these outcomes. We evaluated whether there was the necessity for an interaction term of depression and anxiety, and we included the interaction term in the model, if the corresponding p-value of the interaction was < 0.05. Results were presented as beta-coefficients and corresponding 95% confidence intervals (95% CI).

For the binary outcomes MCID at 24 months for SSM symptom and SSM function, multiple logistic regression models (adjusted for the same confounders) were used to estimate odds ratios (OR) and the corresponding 95% CI. In all multiple regression models, missing values were excluded listwise.

### Sensitivity analysis

A common concern in observational research is the presence of unmeasured confounding. Unmeasured confounding could affect the estimate of the variable of interest in principle in both directions (in- or decrease) of the estimated effect. In a first step, we adjusted the estimated effects of depression and anxiety for a set of eight pre-defined confounders. Because we could not exclude that other unmeasured confounders affect the results, we used a benchmarking method recently proposed by Cinelly and Hazlett^16^. We used a multiple linear model to assess the robustness of the estimated effects of baseline depression/anxiety on continuous outcomes SSM symptoms/function at 24 months. The idea was to quantify and assess visually how strong an unmeasured confounder would have to be to explain away the original effect^16^. In our study, the adjusted estimated effects of depression and anxiety from multiple linear mixed-effects regression models was benchmarked against gender, representing the most relevant confounder of clinical importance in this context, if there was strong evidence for an association. The proposed method used contour plots of the original estimate against its reduction in the direction of no effect with the magnitude of multiples of the gender effect. Estimated effects that are robust up to multiple times the benchmarking variable are considered less likely to be explained away by unmeasured confounding.

All analyses were performed with the statistical software R^27^ in combination with dynamic reporting.

## Results

### Patient characteristics

**Figure 1:**
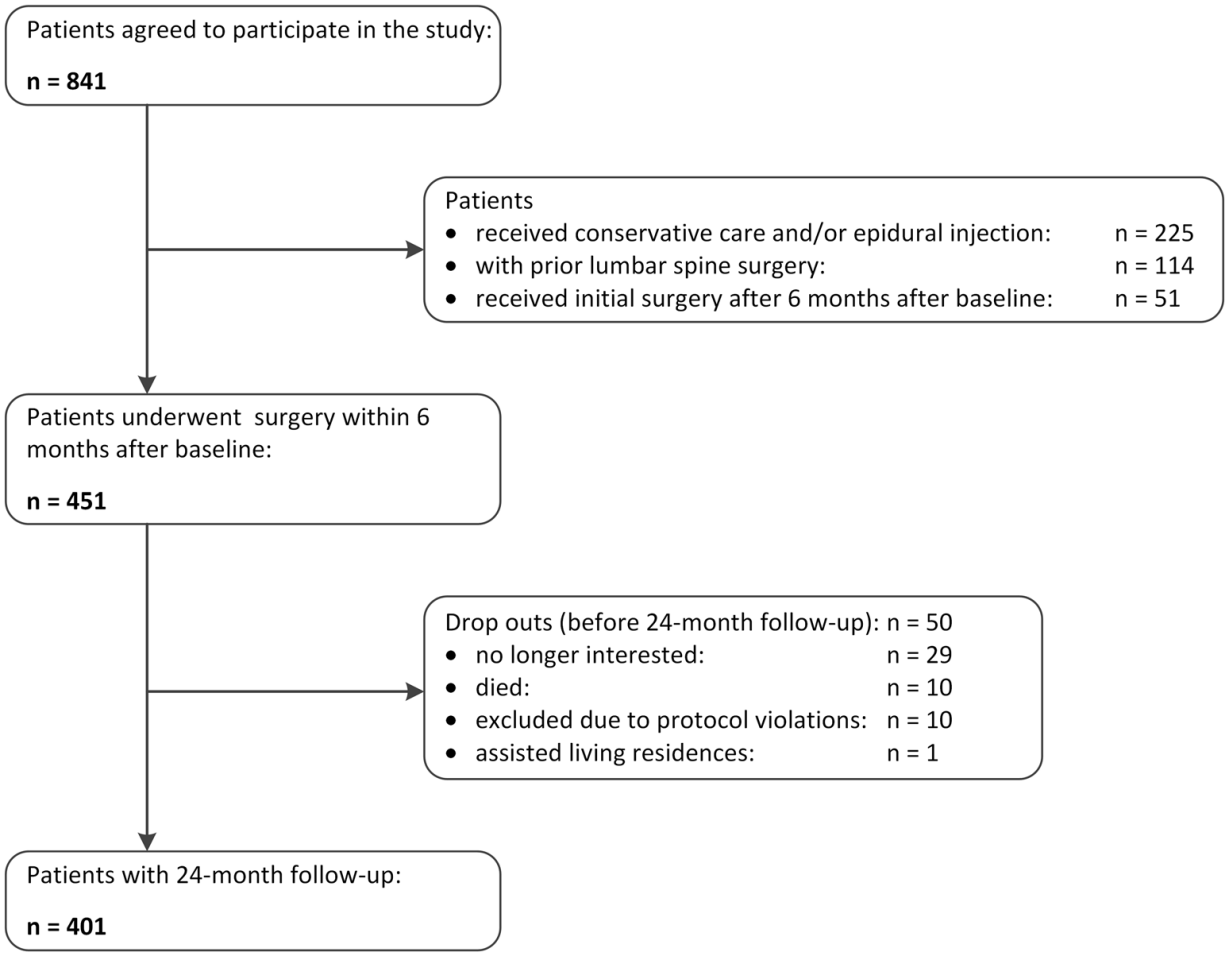
Study flow.

Out of 451 patients who were eligible for the current study, 401 patients (88.91%) completed their 24-month follow-up assessment after surgery and were included in the analysis (Fig. 1). Reasons for missing follow-up evaluations included drop-out or moving to a residential home (n = 30, 6.65%), death (n = 10, 2.22%), and exclusion due to protocol violations (n = 10, 2.22%).

In total 50.37% were female, the mean age was 72.50 years (SD 8.41), mean BMI 27.30 kg/m^2^ (SD 4.61), and symptom duration was > 6 months in 76.25% (Table 1). Compared to patients without depressive symptoms, depressed patients (n = 72, 17.95%) were more often women (63.89% vs. 47.42%) and had a higher mean score in the HADS anxiety subscale (8.49 vs. 3.69). Compared to patients without anxiety, patients with anxiety (n = 80, 19.05%) were younger (mean age 70.54 vs. 72.98 years), more likely female (63.75% vs. 47.04%), had more buttocks pain (88.75% vs 76.64%), complained about more worsening symptoms during the last three months (90.0% vs. 76.88%) and had higher mean score in the HADS depression subscale (8.15 vs. 3.81). Although not statistically significant, the proportion of decompression surgery only was higher in patients with depression compared to non-depressed patients. The proportion of fusion surgery was somewhat higher in patients with anxiety (31.25% vs. 21.50%, p = 0.09).

**Table 1:** Baseline characteristics of the study population.

Variable	Overall	No depression^§^	Depression	Exploratory p-values	No anxiety^‡^	Anxiety	Exploratory p-values
Number of patients (%)	401	329 (82.05)	72 (17.95)		321 (80.05)	80 (19.05)	
Age, mean (SD)	72.50 (8.41)	72.56 (8.19)	72.22 (9.44)	0.76	72.98 (8.23)	70.54 (8.92)	0.02
Female, n (%)	202 (50.37)	156 (47.42)	46 (63.89)	0.02	151 (47.04)	51 (63.75)	0.01
BMI kg/m^2^, mean (SD)	27.30 (4.61)	27.41 (4.60)	26.78 (4.66)	0.29	27.40 (4.68)	26.92 (4.36)	0.41
BMI ≥ 25 kg/m^2^, n (%)	269 (67.08)	223 (67.78)	46 (63.89)	0.62	213 (66.36)	56 (70.00)	0.63
Civil risk*, n (%)	133 (33.17)	105 (31.91)	28 (38.89)	0.32	109 (33.96)	24 (30.00)	0.59
Compulsory education, n (%)	101 (25.19)	85 (25.84)	16 (22.22)	0.62	79 (24.61)	22 (27.50)	0.70
Back pain, n (%)	344 (86.22)	278 (85.02)	66 (91.67)	0.20	271 (84.95)	73 (91.25)	0.20
Buttocks pain, n (%)	317 (79.05)	257 (78.12)	60 (83.33)	0.41	246 (76.64)	71 (88.75)	0.03
Duration of symptoms > 6 months, n (%)	305 (76.25)	250 (76.22)	55 (76.39)	1.00	244 (76.25)	61 (76.25)	1.00
**Problem getting better/worse during last 3 months, n (%)**				0.16			0.03
Getting better	22 (5.50)	19 (5.79)	3 (4.17)		20 (6.25)	2 (2.50)	
Staying about the same	58 (14.50)	53 (16.16)	5 (6.94)		53 (16.56)	5 (6.25)	
Getting worse	318 (79.50)	254 (77.44)	64 (88.89)		246 (76.88)	72 (90.00)	
Don’t know	2 (0.50)	2 (0.61)	0 (0.00)		1 (0.31)	1 (1.25)	
CIRS, mean (SD)	9.32 (3.93)	9.12 (3.97)	10.24 (3.67)	0.40	9.37 (4.03)	9.15 (3.55)	0.73
Diabetes, n (%)	47 (11.72)	36 (10.94)	11 (15.28)	0.73	39 (12.15)	8 (10.00)	0.19
Smoker, n (%)	64 (16.00)	51 (15.55)	13 (18.06)	< 0.001	47 (14.64)	17 (21.52)	< 0.001
HADS anxiety subscale, mean (SD)	4.55 (3.52)	3.69 (2.80)	8.49 (3.80)	< 0.001	3.19 (2.20)	10.03 (2.31)	< 0.001
HADS depression subscale, mean (SD)	4.67 (3.36)	3.44 (2.03)	10.31 (2.35)	< 0.001	3.81 (2.68)	8.15 (3.58)	< 0.001
Epidural injections within 90 days before baseline, n (%)	129 (32.17)	103 (31.31)	26 (36.11)	0.50	99 (30.84)	30 (37.50)	0.31
**Surgery**				0.18			0.09
Decompression surgery only, n (%)	307 (76.56)	247 (75.08)	60 (83.33)		252 (78.50)	55 (68.75)	
Decompression with fusion, n (%)	94 (23.44)	82 (24.92)	12 (16.67)		69 (21.50)	25 (31.25)	

*IQR* interquartile range, *CIRS* cumulative illness rating scale (range from 0 points (best) to 56 points (worst)), *HADS* hospital anxiety and depression scale (each subscale range from 0 points (best) to 21 points (worst)), *SSM* spinal stenosis outcome measure.

^§^Depression defined as HADS depression subscale score ≥ 8 (score-range of 0 (best) to 21 (worst)).

^‡^Anxiety defined as HADS anxiety subscale score ≥ 8 (score-range of 0 (best) to 21 (worst)).

*Living alone, or single/divorced/widowed and living in a nursing/residential home.

χ^2^ and t-tests were used for group comparison.

### Surgical treatment types, surgical complications, and reoperations

Surgical procedures included decompression alone (n = 307, 76.56%) and decompression and fusion surgery (n = 94, 23.44%). Table 2 summarizes intra- and postoperative complications and reoperations. Intra- and postoperative complications occurred in 70 (17.46%) index operations with dural tear (6.22%) being the most common intraoperative complication. There was no difference in complication rates, reoperation rates, and reasons for reoperation in patients with/without depression and anxiety (Table 2).

**Table 2:** Surgical complications, and reoperations.

Variable	Overall	No depression	Depression	Exploratory p-values	No anxiety	Anxiety	Exploratory p-values
n	401	329	72		321	80	
**Complications**							
Intraoperative bleeding	4 (1.00)	3 (0.91)	1 (1.39)	1.00	4 (1.25)	0 (0.00)	0.71
Intraoperative dural tear	25 (6.22)	24 (7.29)	1 (1.39)	0.11	25 (7.79)	0 (0.00)	0.02
Postoperative wound infection	5 (1.24)	3 (0.91)	2 (2.78)	0.48	3 (0.93)	2 (2.50)	0.57
Postoperative osseous infection	1 (0.25)	1 (0.30)	0 (0.00)	1.00	1 (0.31)	0 (0.00)	1.00
Postoperative other*	35 (8.71)	26 (7.90)	9 (12.50)	0.31	26 (8.10)	9 (11.25)	0.50
Number of reoperations: n	53	34	19		37	16	
1 reoperation, patients: n (%)	42 (79.25)	28 (82.35)	14 (73.68)	0.37	30 (81.08)	12 (75.00)	0.63
2 reoperations, patients: n (%)	10 (18.87)	6 (17.65)	4 (21.05)		6 (16.22)	4 (25.00)	
3 reoperations, patients: n (%)	1 (1.89)	0 (0.00)	1 (5.26)		1 (2.70)	0 (0.00)	
Type of reoperation: fusion	32 (60.38)	19 (55.88)	13 (68.42)	0.55	21 (56.76)	11 (68.75)	0.61
Indication: restenosis	38 (71.70)	22 (64.71)	16 (84.21)	0.23	26 (70.27)	12 (75.00)	0.99
Indication: infection	1 (1.89)	1 (2.94)	0 (0.00)	1.00	0 (0.00)	1 (6.25)	0.66
During the 1. year	36 (67.92)	21 (61.76)	15 (78.95)	0.33	26 (70.27)	10 (62.50)	0.81
During the 2. year	16 (30.19)	12 (35.29)	4 (21.05)	0.44	10 (27.03)	6 (37.50)	0.66
During the 3. year	1 (1.89)	1 (2.94)	0 (0.00)	1.00	1 (2.70)	0 (0.00)	1.00
Median days baseline operation to 1. reoperation, (IQR)	199.50 [92.25, 469.75]	213.00 [56.00, 483.50]	193.50 [152.50, 415.00]	0.94	190.00 [56.00, 441.25]	369.50 [153.50, 473.25]	0.20
Median days 1. reoperation to 2. reoperation, (IQR)	200.50 [81.75, 322.75]	207.00 [113.00, 374.50]	149.00 [12.50, 294.50]	0.29	288.50 [159.75, 374.50]	63.50 [12.50, 166.25]	0.14
Median days 2. Reoperation to 3. reoperation, (IQR)	86.00 [86.00, 86.00]	NA [NA, NA]	86.00 [86.00, 86.00]	NA	86.00 [86.00, 86.00]	NA [NA, NA]	NA

*NA* not applicable.

*Example, urosepsis, hemorrhage, wound healing deficit.

### Improvement in symptoms and function between baseline and 24-month

Table 3 summarizes the mean SSM symptoms and function scores at baseline, 12- and 24-months follow-up. Depressed patients expressed higher SSM symptoms compared to patients with no depression (3.39 vs. 3.08, p < 0.001) and function scores (2.55 vs. 2.18, p < 0.001). Baseline anxiety was associated with higher SSM symptoms (3.42 vs. 3.07, p < 0.001) and function scores (2.51 vs. 2.18, p < 0.001).

**Table 3:** Symptoms and function at baseline and follow-up.

Variable	Overall	No depression	Depression	No anxiety	Anxiety
**SSM symptoms, mean (SD)**					
Baseline	3.14 (0.59)	3.08 (0.58)	3.39 (0.59)	3.07 (0.59)	3.42 (0.52)
12-months	2.10 (0.80)	1.99 (0.74)	2.61 (0.84)	2.02 (0.77)	2.38 (0.85)
24-months	2.15 (0.84)	2.05 (0.82)	2.63 (0.77)	2.09 (0.84)	2.41 (0.81)
**SSM function, mean (SD)**					
Baseline	2.24 (0.66)	2.18 (0.64)	2.55 (0.64)	2.18 (0.66)	2.51 (0.58)
12-months	1.52 (0.60)	1.43 (0.53)	1.91 (0.73)	1.49 (0.59)	1.64 (0.64)
24-months	1.55 (0.62)	1.46 (0.56)	1.93 (0.70)	1.52 (0.62)	1.64 (0.61)

*SSM* spinal stenosis outcome measure.

On average, patients improved over time in both outcome scores (Tables 3, 4). Baseline depression was associated with worse SSM symptoms (β = 0.36, 95% CI 0.20 to 0.51, p < 0.001, Table 4a) and SSM function (β = 0.37, 95% CI 0.24 to 0.50, p < 0.001, Table 4b) scores at 24-month follow-up. Baseline anxiety was associated with worse SSM symptoms (β = 0.18, 95% CI 0.03 to 0.33, p = 0.02) without evidence for an association with SSM function (β = 0.03, 95% CI − 0.09 to 0.16, p = 0.63). Whereas each effect size was smaller than the 0.48 points for SSM symptoms and 0.52 points for SSM function score, effects of anxiety and depression combined were clinically relevant. There was no significant interaction between anxiety and depression for SSM symptoms (p = 0.48) and function (p = 0.47).

**Table 4:** Summary of the multiple linear mixed effects regression models for SSM symptom and function at 12 and 24 months in patients with depression and anxiety.

Variable	Estimate	Lower bound of 95% CI	Upper bound of 95% CI	p-value
**(A) SSM symptoms**				
(Intercept)	2.28	1.76	2.81	** < 0.001**
Depression^§^	0.36	0.20	0.51	** < 0.001**
Anxiety^‡^	0.18	0.03	0.33	**0.02**
Time 12 months follow-up	− 1.04	− 1.12	− 0.96	** < 0.001**
Time 24 months follow-up	− 0.99	− 1.07	− 0.91	** < 0.001**
Age	0.003	− 0.004	0.01	0.34
Female	0.20	0.08	0.32	**0.001**
BMI ≥ 25 kg/m^2^	0.14	0.02	0.25	**0.02**
Civil risk*	− 0.02	− 0.15	0.10	0.71
Duration of symptoms > 6 months	0.07	− 0.06	0.20	0.28
CIRS	0.03	0.02	0.05	** < 0.001**
Epidural injections within 90 days before baseline	− 0.02	− 0.13	0.10	0.76
Fusion surgery	− 0.06	− 0.19	0.07	0.39
**(B) SSM function**				
(Intercept)	1.48	1.05	1.92	** < 0.001**
Depression^§^	0.37	0.24	0.50	** < 0.001**
Anxiety^‡^	0.03	− 0.09	0.16	0.63
Time 12 months follow-up	− 0.73	− 0.79	− 0.66	** < 0.001**
Time 24 months follow-up	− 0.70	− 0.76	− 0.63	** < 0.001**
Age	0.005	− 0.001	0.01	0.12
Female	0.19	0.10	0.29	** < 0.001**
BMI ≥ 25 kg/m^2^	0.19	0.10	0.29	** < 0.001**
Civil risk*	− 0.03	− 0.14	0.07	0.54
Duration of symptoms > 6 months	0.01	− 0.10	0.11	0.91
CIRS	0.02	0.01	0.03	**0.01**
Epidural injections within 90 days before baseline	0.03	− 0.07	0.13	0.55
Fusion surgery	− 0.12	− 0.23	− 0.01	**0.03**

A negative value corresponds to reduction of symptom severity/functional disability.

*CIRS* cumulative illness rating scale (range from 0 points (best) to 56 points (worst)).

^§^Depression defined as HADS depression subscale score ≥ 8 (score-range of 0 (best) to 21 (worst)).

^‡^Anxiety defined as HADS anxiety subscale score ≥ 8 (score-range of 0 (best) to 21 (worst)).

*Living alone, or single/divorced/widowed and living in a nursing/residential home.

Significance values are given in bold.

### Sensitivity analysis

**Figure 2:**
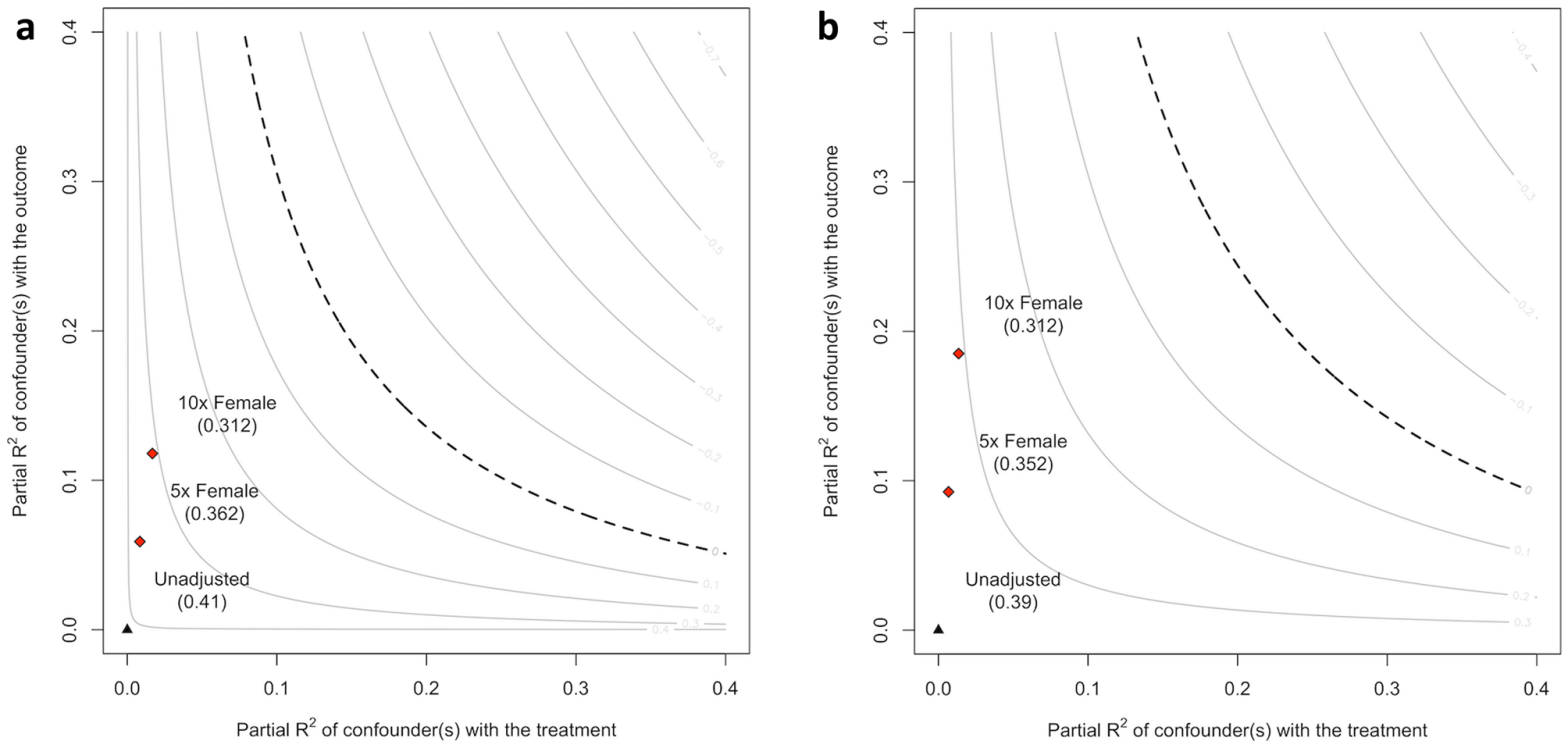
(**a**) Contour plot of robustness of depression for SSM symptoms. The partial R^2^ gives the percentage of variation in a multiple linear model that cannot be explained by the confounders. The unadjusted estimate 0.41 represents the estimate for depression in a multiple linear model with SSM symptoms as outcome, and with the confounders age, gender female, BMI ≥ 25, civil risk, duration of symptoms > 6 months, CIRS, epidural injections within 90 days before baseline, and necessity of fusion surgery. The estimate of 0.36 represents the estimate for depression on SSM symptoms, if there was an unmeasured confounders with the strength of association of five times female gender present. Accordingly, the estimate of 0.31 shows the estimate for depression on SSM symptom if there was an unmeasured confounder with the strength of association of ten times gender female present. The sensitivity analysis shows that even in the presence of a strong unmeasured confounder, the estimate of depression on SSM symptom would remain robust. (**b**) Contour plot of robustness of depression for SSM function. The partial R2 gives the percentage of variation in a multiple linear model that cannot be explained by the confounders. The unadjusted estimate 0.39 represents the estimate for depression in a multiple linear model with SSM function as outcome, and with the confounders age, gender female, BMI ≥ 25, civil risk, duration of symptoms > 6 months, CIRS, epidural injections within 90 days before baseline, and necessity of fusion surgery. The estimate of 0.35 represents the estimate for depression on SSM function, if there was an unmeasured confounders with the strength of association of five times female gender present. Accordingly, the estimate of 0.31 shows the estimate for depression on SSM function if there was an unmeasured confounder with the strength of association of ten times gender female present. The sensitivity analysis shows that even in the presence of a strong unmeasured confounder, the estimate of depression on SSM function would remain robust.

The sensitivity analysis was performed for depression, as there was strong evidence for an association with SSM symptoms and SSM function. The sensitivity analysis revealed that the estimated effect of depression on SSM symptoms and SSM function was robust even if an unmeasured confounder of the magnitude of at least 10 times gender was present in our study (Fig. 2a,b).

### Clinical relevance of depression and anxiety

The number of patients with clinically meaningful improvement (MCID) in SSM symptoms was 280 (69.83%; depression 63.89%, no depression 71.12%, anxiety 68.75%, and no anxiety 70.09%) over 2 years follow-up. The corresponding numbers for patients with MCID in SSM function were 254 (63.34%; depression 56.94%, no depression 64.74%, anxiety 67.50%, and no anxiety 62.31%). There was no evidence that depression was associated with decreased odds for a MCID in SSM symptoms (OR 0.70, 95% CI 0.37 to 1.34, p = 0.28) and function (OR 0.59, 95% CI 0.32 to 1.11, p = 0.10) at 24-month follow-up. There was also no evidence that anxiety was associated with a decreased odds for a MCID in SSM symptoms (OR 1.01, 95% CI 0.54 to 1.96, p = 0.97) and SSM function (OR 1.47, 95% CI 0.80 to 2.79, p = 0.22) scores at 24-month follow-up (Table 5).

**Table 5:** Summary of the multiple logistic regression models for minimal clinical important difference (MCID) in the SSM symptoms and function at 24 months in patients with depression, anxiety.

Variable	OR**	Lower bound of 95% CI	Upper bound of 95% CI	p
**(A) SSM symptoms**				
Depression^§^	0.70	0.37	1.34	0.28
Anxiety^‡^	1.01	0.54	1.96	0.97
Age	0.97	0.94	0.10	**0.02**
Female	1.00	0.61	1.66	1.00
BMI ≥ 25 kg/m^2^	0.77	0.46	1.27	0.31
Civil risk*	0.76	0.45	1.28	0.29
Duration of symptoms > 6 months	0.55	0.30	0.97	**0.04**
CIRS	0.98	0.92	1.04	0.44
Epidural injections within 90 days before baseline	1.69	1.02	2.85	**0.05**
Fusion surgery	1.02	0.58	1.81	0.96
**(B) SSM function**				
Depression^§^	0.59	0.32	1.11	0.10
Anxiety^‡^	1.47	0.80	2.79	0.22
Age	0.97	0.94	1.00	0.05
Female	0.87	0.54	1.39	0.55
BMI ≥ 25 kg/m^2^	0.70	0.44	1.12	0.14
Civil risk*	0.94	0.57	1.56	0.81
Duration of symptoms > 6 months	0.47	0.27	0.82	**0.01**
CIRS	0.95	0.90	1.00	0.07
Epidural injections within 90 days before baseline	1.56	0.98	2.53	0.07
Fusion surgery	0.84	0.50	1.43	0.52

^§^Depression defined as HADS depression subscale score ≥ 8 (score-range of 0 (best) to 21 (worst)).

^‡^Anxiety defined as HADS anxiety subscale score ≥ 8 (score-range of 0 (best) to 21 (worst)).

*Living alone, or single/divorced/widowed and living in a nursing/residential home.

**A value smaller than 1 corresponds to a reduced chance to achieve minimal important difference of SSM symptoms/function subscale.

Significance values are given in bold.

## Discussion

In this study of 401 patients undergoing surgery for symptomatic DLSS, we found a robust association between baseline depression with more severe symptoms and worse function even after accounting for the potential presence of strong unmeasured confounding. Although the effect of anxiety alone was clinically not relevant, baseline depression and anxiety combined were associated with a clinically relevant increase in the severity of symptoms over the study period. Baseline depression and anxiety did not reduce the odds for a clinical meaningful improvement in symptoms and function scores and were not associated with complication rates and reoperation.

### Results in the light of existing literature

The results from our study expands on the conclusion of a systematic review published in 2014^13^. Although our study supports the authors conclusion that depression is a prognostic indicator in patients undergoing surgery for lumbar spinal stenosis^13^ for symptoms and function, depression did not result in a reduced odds for a clinical meaningful improvement after surgery. Lebow et al.^28^ argued that pain from disc herniation might contribute to preoperative depression and anxiety and that discectomy improves mental health and well-being during the postoperative period over months with improvement in anxiety and depression. Three studies showed level of depression measured preoperatively did not predict the surgical outcome^15,28,29^ and that depression improved postoperatively in patients with a favorable outcome and did not improve in patients with a poor treatment outcome, suggesting that psychological disorders may be the result of DLSS related complaints^29,30^. However, other studies found preoperative depression to be associated with poorer life satisfaction after surgery^31^ and poorer treatment outcome (symptom severity, disability and walking capacity) after surgery^31,32^. Whether depression alone or as a result of a general life-dissatisfaction, as suggested by a study following patients over 10 years after surgery for DLSS^33^, warrants further investigation to elucidate whether preoperative depression should be addressed as a modifiable factor.

A systematic review concluded that anxiety and catastrophizing may be a relevant and modifiable factor to consider, in particular, in musculoskeletal surgery^34^. In one study preoperative anxiety was associated with postoperative anxiety and physical complaints after lumbar spinal surgery^35^. Pain related anxiety and avoidance behavior are physiological responses after an acute incidence. However, over time avoidance may result in disuse and disability and may increase the disease burden^36,37^. Therefore, anxiety and avoidance may be potentially modifiable coping styles^38–40^ and relevant to consider after spine surgery. Indeed, patients with persistent fear avoidance beliefs after decompression surgery for DLSS reported poorer treatment outcome after 1 year than patients with decreased or no fear avoidance beliefs after surgery^41,42^. In the current study, we observed an additive effect of anxiety to depression which reached clinical relevance for symptoms which may warrant further studies.

### Implications for research

Depression and anxiety may be relevant and modifiable factors to consider during the postoperative phase and should be further studied. In particular, in patients with persistent depression despite improvement after surgery, concomitant psychological interventions should be studied. Further, the importance of pre- and perioperative stress on postoperative complications is not well understood. Starkweather et al.^43^ showed that psychological stress had an immunosuppressive effect that may result in an increased risk for complications.

### Implications for clinical practice

Although patients with preoperative depression and anxiety may report more symptoms after a 2-year follow-up, they are equally likely to experience a clinical meaningful improvement as patients without depression or anxiety.

### Strength and limitations

A strength of the study include the large number of patients undergoing in surgery for symptomatic DLSS, the prospective recording of known prognostic factors, and the use of a novel method to benchmark the estimated effect of depression against the clinically most relevant confounder gender. A strength of our study is that we used new methods to assess the relevance of the findings accounting for unmeasured confounding. There are several limitations to discuss. First, depression and anxiety were based on self-reported measures (the validated HADS questionnaire) and we had no information on the clinical diagnosis and treatment of depression. Therefore, we were not able to determine the severity and nature of depression and anxiety. Further, we do not know whether some patients received treatment for depressive symptoms that may influent treatment outcome. Second, we did not collect information on anxiety and depression during the follow-up period. Therefore, we were not able to assess the interaction between treatment, depression/anxiety, and outcome.

## Conclusion

In patients undergoing surgery for symptomatic DLSS, we observed a robust association between preoperative depression and more symptoms and disability at 12- and 24-months. Anxiety was associated with more symptoms at 12- and 24-months but the effect was small. Both, baseline depression and anxiety did not influence the odds for clinically meaningful improvement in symptoms and function scores.

## Supplementary Information

Supplementary Information.

## Abbreviations

DLSS: Degenerative lumbar spinal stenosis
LSOS: Lumbar Stenosis Outcome Study
HADS: Hospital anxiety and depression scale
CIRS: Cumulative illness rating scale
SSM: Spinal stenosis measure
MCID: Minimal clinically important difference
