# Supplementary material for: Association between depression and anxiety on symptom and function after surgery for lumbar spinal stenosis
Source: Sci Rep. 2022 Feb 18;12:2821. doi: 10.1038/s41598-022-06797-1 (PMC8857319; doi:10.1038/s41598-022-06797-1)
Supplement: Supplementary file 1 — Supplementary Information. [file 41598_2022_6797_MOESM1_ESM.docx]

**Supplementary File**

**Appendix 1: German Version of the Spinal Stenosis Measure (SSM)**

1a SSM Symptom Subscale:

Beantworten Sie bitte folgende Fragen bezogen auf den letzten Monat:

1. Wie würden Sie die Schmerzen beschreiben, die Sie durchschnittlich im Rücken, im Gesäss und ausstrahlend in die Beine verspürt haben?

keine bis sehr starke Schmerzen

2. Wie oft hatten Sie Schmerzen im Rücken, im Gesäss oder in den Beinen?

weniger als einmal pro Woche bis täglich, ununterbrochen

3. Wie würden Sie die Schmerzen in Ihrem Rücken oder im Gesäss beschreiben?

keine Schmerzen bis sehr starke Schmerzen

4. Wie würden Sie die Schmerzen in Ihren Beinen und Füssen beschreiben?

keine Schmerzen bis sehr starke Schmerzen

5. Hatten Sie Taubheitsgefühle oder Ameisenkribbeln in Ihren Beinen oder Füssen?

gar nicht bis sehr stark

6. Stellten Sie eine Muskelschwäche in Ihren Beinen oder Füssen fest?

gar nicht bis sehr stark

7. Litten Sie unter Gleichgewichtsstörungen?

Keine bis oft oder das Gefühl, keinen sicheren Stand zu haben

1b SSM Function Scale Subscale

8. Wie weit konnten Sie am Stück laufen?

Mehr als 3 km bis weniger als 15 m

9. Sind Sie zum Vergnügen draussen spazieren gegangen?

10. Haben Sie Lebensmittel- oder andere Einkäufe erledigen können? Könnten Sie Lebensmittel- oder andere Einkäufe erledigen, wenn Sie müssten?

11. Konnten Sie in den Wohnräumen Ihres Hauses oder Ihrer Wohnung umher laufen?

12. Konnten Sie von Ihrem Schlafzimmer ins Bad laufen?

Antworten problemlos bis nein

2c SSM Satisfaction Subscale

In den folgenden Fragen werden Sie nach Ihrer Zufriedenheit mit der Therapie gefragt. Therapie kann bedeuten dass Sie operiert wurden, Medikamente erhielten oder eine Physiotherapie verordnet bekamen. Keine Therapie kann medizinisch gesehen auch eine Therapie sein. Bitte beantworten Sie die Fragen in jedem Falle, auch wenn Sie keine Therapie erhielten. Beantworten Sie bitte folgende Fragen bezogen auf den letzten Monat.

1. dem Gesamtergebnis Ihrer Operation /der Therapie ?

2. dem Rückgang der Schmerzen nach der Operation /der Therapie ?

3. Ihrem Gehvermögen nach der Operation /der Therapie

4. Ihrer Fähigkeit, Haus- und Gartenarbeiten sowie Tätigkeiten am Arbeitsplatz zu verrichten?

5. Ihrer Kraft in den Oberschenkeln, Beinen und Füssen ?

6. Ihrem Gleichgewichtssinn oder der Stabilität in Ihren Füssen ?

Antworten: Sehr zufrieden bis sehr unzufrieden
